# Bioavailability of Cadmium in Inexpensive Jewelry

**DOI:** 10.1289/ehp.1003011

**Published:** 2011-03-04

**Authors:** Jeffrey D. Weidenhamer, Jennifer Miller, Daphne Guinn, Janna Pearson

**Affiliations:** Department of Chemistry, Geology & Physics, Ashland University, Ashland, Ohio, USA

**Keywords:** cadmium, children’s health, import safety, jewelry, potential cadmium exposures

## Abstract

Objectives: We evaluated the bioavailability of Cd in 86 components of 57 jewelry items found to contain high levels of Cd (> 10,000 ppm) by X-ray fluorescence (XRF), using extractions that simulate mouthing or swallowing of jewelry items.

Methods: We screened jewelry for Cd content by XRF. Bioavailability was measured in two ways. Items were placed in saline solution at 37°C for 6 hr to simulate exposures from mouthing of jewelry items. Items were placed in dilute hydrochloric acid (HCl) at 37°C for 6–96 hr, simulating the worst-case scenario of a child swallowing a jewelry item. Damaged pieces of selected samples were also extracted by both methods to determine the effect of breaching the outer plating on bioavailability. Total Cd content of all items was determined by atomic absorption.

Results: The 6-hr saline extraction yielded as much as 2,200 µg Cd, and 24-hr dilute HCl extraction yielded a maximum of > 20,000 µg Cd. Leaching of Cd in dilute HCl increased linearly over 6–96 hr, indicating potential for increasing harm the longer an item remains in the stomach. Damage to jewelry by breaching the outer plating generally, but not always, increased Cd release. Bioavailability did not correlate directly with Cd content.

Conclusions: These results indicate the potential for dangerous Cd exposures to children who wear, mouth, or accidentally swallow high-Cd jewelry items.

Recent news reports have highlighted the emerging problem of cadmium (Cd) contamination of children’s and other inexpensive jewelry imported from China (Hoshiko and Olesen 2010; Pritchard 2010; Pritchard and Donn 2010). The use of high concentrations of Cd in inexpensive jewelry is attributed to more stringent regulations of lead content and to declining costs for Cd derived from nickel-Cd batteries as these are phased out and replaced with more environmentally benign products (Hoshiko and Olesen 2010). Although there are no regulations in the United States on the Cd content of children’s jewelry, the U.S. Consumer Product Safety Commission (CPSC) has now issued five recalls of jewelry for Cd contamination (U.S. CPSC 2010a, 2010b, 2010d, 2010f, 2010g), and the chair of the CPSC has advised parents to take all inexpensive jewelry away from children because of the potential risks (U.S. CPSC 2010c).

The magnitude of the potential problem is difficult to estimate, although inexpensive jewelry marketed to children is widely sold in the United States. CPSC staff have previously reported (U.S. CPSC 2006), based on analysis of National Electronic Injury Surveillance System data, that from 2000 to 2005 there were > 300,000 emergency room visits by children ≤ 18 years of age for foreign object ingestion. It was further estimated that 80% of these children were < 7 years of age and that approximately 20,000 of the swallowed objects were jewelry items. Swallowing is not the only potential route of exposure to heavy metals in jewelry. In 2008, an Illinois infant was lead poisoned by mouthing her mother’s key chain, resulting in the recall of 51,000 key chains by Wal-Mart (U.S. CPSC 2008).

The toxicity of Cd is well known. Primary concerns with chronic exposure include osteotoxicity (Järup and Alfven 2004; Satarug et al. 2010) and kidney damage (Noonan et al. 2002; Satarug et al. 2005, 2010). Epidemiologic evidence suggests that certain populations, notably diabetics, are more susceptible to the toxic effects of Cd (Nordberg et al. 2009). At low doses, Cd exposure induced a basal-like cancer phenotype in immortalized but nontumorigenic cells derived from normal human breast epithelium (Benbrahim-Tallaa et al. 2009).

The major sources of Cd exposure in the general population include food and tobacco smoke (Satarug and Moore 2004). Any increase in chronic Cd intake due to new exposure sources is of concern because Cd bioaccumulates, with a half-life in the kidney of 10–30 years [U.S. Environmental Protection Agency (EPA) 1997]. In a recent review, Järup and Åkesson (2009) pointed out that a significant fraction of the adult nonsmoking population has urinary Cd > 0.5 µg/g creatinine and that measurable effects of Cd exposure at this level can be seen on bone, in early markers of kidney damage, and increased cancer risk. They concluded that “this implies no margin of safety” between current levels of exposure and levels that cause adverse effects.

In October 2010, the CPSC issued a report recommending that the ASTM International Subcommittee on Toy Safety develop new standards for Cd in children’s jewelry (U.S. CPSC 2010e) based on two measurements of Cd bioavailability. The recommended maximum exposure limits are *a*) 18 µg Cd for a 6-hr saline extraction to simulate mouthing of items, and *b*) 200 µg Cd for a 24-hr extraction in dilute hydrochloric acid (HCl) to simulate ingestion of an object by a child (U.S. CPSC 2010e). Industry groups favor a shorter, 2-hr extraction period with dilute acid, as is used in European standard EN-71 (Mead 2010). The CPSC recommendation was derived from an analysis of the Agency for Toxic Substances and Disease Registry (ATSDR) chronic duration oral minimum risk level of 0.1 µg/kg body weight (BW)/day (ATSDR 2008). The ATSDR minimum risk level is a factor of 10 lower than the 1 µg/kg BW/day reference dose recommended by the U.S. EPA (1997), but neither of these guidelines were specifically established for children. Greater intestinal absorption of Cd in children compared with adults would imply that use of the adult guidelines could result in underestimation of risk in children. An enhanced rate of Cd absorption in children (particularly girls) has recently been revealed from modeling of Cd exposures in the general U.S. population (Ruiz et al. 2010). Schoeters et al. (2006), in a review of the health effects of Cd on children, concluded that Cd exposure and accumulation early in life can result in kidney damage and osteoporosis later in life. They urged that Cd exposure in children be reduced as much as is feasible both to prevent toxic effects of Cd to children and to prevent the bioaccumulation of Cd that may have effects that only show up decades later. Only one previous report (Streicher-Porte et al. 2008) provides data on the bioavailability of Cd from high-Cd jewelry. Given the paucity of data and the apparent increase in the prevalence of Cd in inexpensive jewelry items, the objective of this study was to characterize the potential for children to be exposed to Cd from Cd-containing jewelry by using extractions that simulate mouthing or swallowing of jewelry items. Cd-contaminated jewelry was identified through the use of X-ray fluorescence (XRF). This technique has gained popularity as a rapid method to screen large numbers of samples for heavy metals (Cox and Green 2010; U.S. CPSC 2009). Because the overall prevalence of Cd-containing jewelry was below 20%, XRF screening was crucial in the identification of high-Cd components for more detailed analysis.

## Materials and Methods

*XRF analysis.* XRF screening of jewelry items was conducted using a Niton XL3t GOLDD XRF spectrometer mounted in a test stand (Thermo Fisher Scientific, Billerica, MA, USA). Samples were analyzed in Testall mode for 60 sec.

*Samples.* Samples were drawn from a collection of 612 inexpensive, imported jewelry items that have been purchased in the United States since 2006. Jewelry items were selected based on price (the maximum price per item was $12 and most items were priced below $5) and an effort to obtain products from a variety of distributors, retailers, and geographic locations. Of these 612 items, XRF screening identified 117 with components containing > 10,000 ppm Cd, 86 containing > 50,000 ppm Cd, and 70 containing > 100,000 ppm (= 10%) Cd. For this study, a total of 69 items with 101 distinct high-Cd components were subjected to further testing. Dilute HCl extraction (to simulate swallowing of jewelry) was carried out on a subset of 86 components from 57 jewelry items. With the analysis of selected duplicates and of damaged duplicates of selected components, a total of 116 samples were analyzed by dilute HCl extraction. Saline extraction (to simulate mouthing of jewelry) was carried out before dilute HCl extraction on a subset of 32 components from 22 jewelry items. With the analysis of selected duplicates and of damaged duplicates of selected components, a total of 48 samples were analyzed by saline extraction. Additional components not subjected to testing of bioavailability were analyzed by XRF and by digestion for total Cd content.

Based on package labeling, 67 of the jewelry items were made in China, one was made in Taiwan, and one was of unknown origin. Items were selected to provide a wide range of total Cd content so that bioavailability could be evaluated in a diverse set of samples, and most items tested were recently purchased (late 2009 and early 2010) because of concern about the emerging nature of this threat to children’s health. In all, the high-Cd samples dated to 2006 (six items), with additional samples from 2008 (six items), and a majority of items that were purchased in late 2009 (11 items) and early 2010 (46 items). Duplicate samples of many of these items were evaluated but are not counted in the above totals. Twenty of the 69 jewelry items tested were readily identifiable by labeling (indicating suitability for children), placement in the children’s section of the store, and/or appearance (children’s characters or themes) as children’s jewelry items. Labeling and/or appearance of some items was ambiguous, and approximately 10–15 items would clearly not be classified as children’s jewelry but were included in the interest of having a larger sample set to evaluate the bioavailability characteristics of high-Cd jewelry.

*Saline-extractable Cd.* Saline extraction has been used by the U.S. CPSC (1997) to simulate exposures obtained by mouthing behaviors. In a shaker bath, samples were suspended in a volume of dilute saline solution equivalent to 50 times the mass of the item and extracted at 37°C for 6 hr. Solutions were then diluted to known volumes and analyzed by atomic absorption (AA). Because of limited quantities of jewelry items, samples selected for saline extraction were, in general, subsequently tested by the 0.07 M HCl extraction. Swallowing an item after mouthing it is a plausible sequence of events, and because the saline extraction is milder than the dilute HCl extraction, any effect of saline extraction on subsequent HCl extraction was expected to be small.

*Dilute HCl-extractable Cd.* This procedure follows the methods prescribed in ASTM F963 (ASTM 2008) as detailed in the CPSC Standard Operating Procedure for determining lead and its bioavailability in children’s metal jewelry (U.S. CPSC 2005b). In a shaker bath, samples were suspended in a volume of dilute HCl (0.07 M) equivalent to 50 times the mass of the item at 37°C to simulate exposure to an item that is swallowed. In initial studies, sequential extraction times of 6, 18, 24, and 48 hr were used to determine the total HCl-extractable Cd for time points of 6, 24, 48, and 96 hr. The 24-hr time point was the sum of the 6-hr and 18-hr extractions, the 48-hr time point was the sum of the 6-, 18-, and 24-hr extractions, and the 96-hr time point was the sum of all sequential extractions. Items were extracted for the specified length of time; the extraction solutions were removed and replaced with fresh solutions, and then extracted for the next time interval to determine Cd accessibility. Because initial studies indicated that the increase in HCl-extractable Cd was linear over 96 hr, 24 hr was used as the time interval for subsequent extractions.

Damage to the outer surface of inexpensive jewelry may be expected to occur through normal use, but the effect of this damage on potential leaching characteristics has not been explored. Most jewelry components were analyzed intact; however, duplicates of selected samples were damaged by breaching the outer plating to examine possible effects on leaching characteristics. Metal cutters were used to make an approximately 1- to 2-mm cut in the jewelry component, which was then subjected to saline or dilute HCl extraction as described above.

*Total Cd.* Total Cd was determined by digestion of duplicate 0.1- to 0.2-g samples in 50% trace metal grade nitric acid, followed by appropriate dilution. For the damaged charms subjected to the saline and dilute HCl extractions, samples for total Cd analysis were removed at a distance from the damaged portion of the charm in case there had been depletion of Cd content at the site of damage. Total Cd concentrations reported are based on the remaining mass after the saline and dilute HCl extractions (if performed).

*AA methods.* Cd concentrations were measured by AA spectrometry (SpectrAA 220 FS; Varian, Walnut Creek, CA) using an air-acetylene flame at 228.8 nm. Calibration was linear over the range of 0–3 mg/L, and calibration standards were prepared from a 1,000 ± 4 mg/L certified reference material Cd standard for AA (no. 51994; Fluka Analytical, St. Louis, MO). Quality assurance was maintained by analysis of blank and fortified samples. All glassware used in these studies was washed with concentrated nitric acid before use. Blanks were clean, indicating effectiveness of these procedures. The average recovery of Cd from fortified standards spiked with 100 µg Cd was 100.5 ± 3.9%. For total Cd, reagent-grade Cd granules served as a reference standard, and analysis of these granules averaged 99.2 ± 1.8% Cd. The AA detection limit for Cd was 0.035 mg/L.

## Results

*Saline-extractable Cd.* Jewelry components were chosen for saline extraction to provide a wide range of Cd content based on estimates from XRF screening of samples. A total of 34 samples were subjected to the 6-hr saline extraction. Additional tests were run on duplicates of 14 samples that were damaged, as described previously, to determine the effect of breaching the outer coating of items. The average total Cd content of these 48 samples (34 undamaged samples, and 14 damaged duplicates) was 28.5%, with a range of 1.4–89.2%. Results for the undamaged samples are summarized in Table 1. Twenty samples yielded < 1.0 µg Cd by this extraction procedure. Nine samples yielded > 18.0 µg Cd, the maximum recently recommended by the CPSC for saline extractions (U.S. CPSC 2010e). Duplicate samples of two charms from a child’s bracelet yielded results both above and below the 18-µg threshold recommended by the CPSC. For the first charm, 11.0 and 27.4 µg saline-extractable Cd were obtained, and the second charm yielded 11.5 and 37.4 µg saline-extractable Cd.

**Table 1:** Summary of results of 6-hr saline extractions of 34*a* undamaged high-Cd jewelry samples.

Table 1. Summary of results of 6-hr saline extractions of 34*a* undamaged high-Cd jewelry samples.	
No. of components	Saline-extractable Cd (µg)
11	< LOD
9	< 1.0
2	1.0–4.9
3	5.0–18.0
2	18.1–49.9
1	50–99
5	100–499
1	> 500
LOD, limit of detection. **a**The 34 samples tested represent 32 distinct jewelry components, with duplicate samples of two components.	

Additional tests were run on duplicates of 14 components that were damaged, as described previously, to determine the effect of breaching the outer coating of items (Table 2). The effect of damage to the jewelry items on saline-extractable Cd was dependent on the sample. Nine of the 14 damaged samples yielded < 1.0 µg saline-extractable Cd. Duplicate samples of a football pendant yielded the highest Cd by this extraction, and damage resulted in a modest increase (2,189 µg) compared with the undamaged sample (2,109 µg). Three samples, two of which were children’s jewelry items, yielded an average of only 1.1 µg saline-extractable Cd when damaged, but the undamaged components yielded an average of 85.8 µg Cd. In each case, total Cd content of the damaged and undamaged components was comparable. The overall correlation between saline-extractable Cd and total Cd was weak (*r*^2^ = 0.35).

**Table 2:** Comparison of results of 6-hr saline extractions for undamaged and damaged duplicates of 14 high-Cd jewelry samples.

Table 2. Comparison of results of 6-hr saline extractions for undamaged and damaged duplicates of 14 high-Cd jewelry samples.				
		Saline-extractable Cd (µg)		
Item		Undamaged		Damaged
1		< LOD		< LOD
2		< LOD		< LOD
3		< LOD		< LOD
4		< LOD		< LOD
5		< LOD		< LOD
6		< LOD		0.68
7		0.18		1.28
8		0.38		0.58
9		0.48		1.38
10		0.78		0.58
11		16.3		1.28
12		100.1		1.08
13		140.9		0.98
14		2,109		2,189
LOD, limit of detection. Samples were damaged by making a small cut in the outer coating, as described in “Methods.”				

*Dilute HCl-extractable Cd over time.* A detailed investigation of the increase in dilute HCl-extractable Cd over time was undertaken with two high-Cd jewelry items for which multiple duplicates were available and which had very different total Cd content. The two jewelry items included a set of three sandal charms labeled as appropriate for children > 3 years of age (total Cd 28.8–51.4%), and a children’s bracelet with heart-shaped charms (total Cd, 89.2–94.8%). Six intact sandal charms and six intact heart-shaped charms were tested by dilute HCl extraction for 6, 24, 48, and 96 hr. An additional six charms from each piece of jewelry were damaged, as described previously, and tested in the same manner.

**Figure 1:**
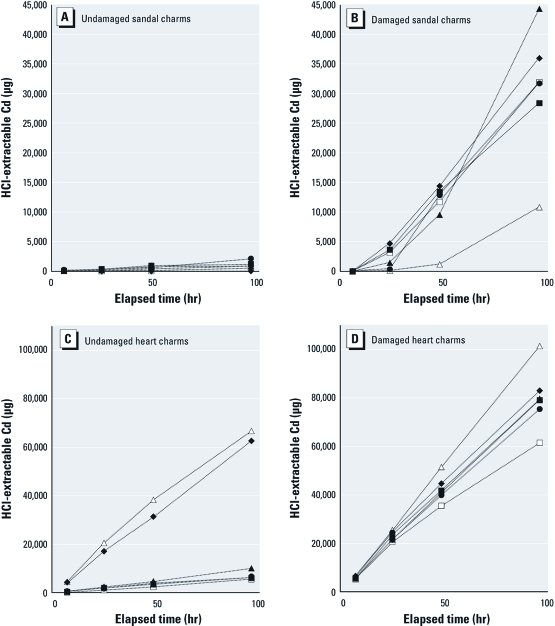
Dilute HCl-extractable Cd measured at 6, 24, 48, and 96 hr for six replicate undamaged and damaged charms from two jewelry items labeled for children > 3 years of age. (*A* and *B*) results for undamaged and damaged sandal charms, respectively; (*C* and *D*) results for undamaged and damaged heart-shaped charms from a child’s charm bracelet.

**Figure 2:**
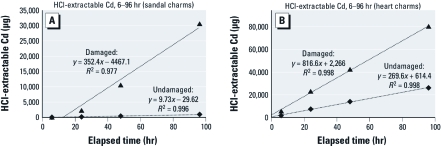
Linear regression data for the mean HCl-extractable Cd over 6–96 hr for charms included in Figure 1.

Results for charms from both jewelry items showed similar trends. Extractions of undamaged charms showed high variability (Figures 1A,C). For the individual sandal charms, results of the dilute HCl extraction ranged at 96 hr from a minimum of 13 µg Cd to almost 2,100 µg Cd, with a mean of 912 µg Cd (Figure 1A). Total Cd content for these charms averaged 37.0 ± 1.5% total Cd, implying that the variation in dilute HCl-extractable Cd was attributable to variations in the quality of surface plating. The CPSC has reported that Cd migration into dilute HCl decreased as zinc content increased (U.S. CPSC 2010e). However, there was no correlation here between zinc content of the sandal charms as measured by XRF, which ranged from 43,900 to 126,700 ppm, and extractable Cd at 96 hr (*r*^2^ = 0.004), In addition, Cd content as measured by XRF and dilute HCl-extractable Cd were not correlated (*r*^2^ = 0.16 at 96 hr for the sandal charms), so the reason for this high variability in Cd bioavailability remains unclear. The heart charms had higher total Cd content (91.2 ± 1.2%) and also showed high variability in dilute HCl-extractable Cd, although with a different pattern. Four charms yielded between 5,650 to 10,020 µg Cd after 96 hr, but two other charms yielded more than 60,000 µg Cd. For both sets of undamaged charms, the mean dilute HCl-extractable Cd for 6–96 hr was highly linear (Figure 2A,B).

Damaging charms greatly increased the amount of Cd released over time in the HCl extractability test (Figure 1B,D), although charm-to-charm variation remained pronounced. The mean dilute HCl-extractable Cd for damaged sandal charms was 30,600 µg Cd after 96 hr and almost 80,000 µg Cd for the heart charms. One of the damaged heart charms yielded 101,400 µg Cd over 96 hr. The damaged sandal charms showed some increase in Cd release rate with time (Figure 2A), whereas for the damaged heart charms, the mean dilute HCl-extractable Cd for 6–96 hr was highly linear (Figure 2B). Two of the undamaged heart charms released Cd in the same range as the damaged charms (Figure 1C). However, none of the charms had been worn, and there was no obvious damage to any of the intact charms upon visual inspection, so the reason for this result is unclear.

*Dilute HCl-extractable Cd: 24-hr measurements.* Based on results showing that the increase in acid-extractable Cd was linear over time, 24 hr was chosen as the standard extraction interval for a larger set of 92 additional samples. Results after 24-hr extraction for the 92 new samples plus six undamaged sandal charms and six heart-shaped charms are summarized in Table 3. The mean total Cd of these 104 samples was 35.9%. Although almost half (46) of the samples yielded < 25 µg Cd over 24 hr, 31 samples from 14 distinct jewelry items exceeded 200.0 µg Cd, the threshold recently recommended by the CPSC for 24-hr dilute HCl extractions (U.S. CPSC 2010e). Fourteen samples yielded > 1,000 µg Cd, and two samples (the football pendant that yielded the largest amount of saline-extracted Cd and one of the heart charms) exceeded 20,000 µg Cd at 26,430 and 20,520 µg Cd, respectively. In general, items with higher total Cd concentration leached the highest amounts of Cd in this test.

**Table 3:** Summary of results of 24-hr accessibility extractions of 104*a* undamaged high-Cd jewelry samples.

Table 3. Summary of results of 24-hr accessibility extractions of 104*a* undamaged high-Cd jewelry samples.				
No. of components		Accessible Cd (µg)		Mean total Cd (%)*b*
10		< LOD		22.6
36		< 25		26.0
15		25–49		27.9
6		50–99		26.9
6		100–200		30.0
11		201–499		36.8
4		500–999		52.8
7		1,000–2,499		70.0
2		2,500–4,999		43.6
2		5,000–9,999		87.2
0		10,000–14,999		–
3		15,000–19,999		36.1
2		> 20,000		89.9
LOD, limit of detection. **a**The 104 samples tested represent 84 distinct jewelry components. Data for the six undamaged sandal charms and six heart-shaped charms in the 96-hr study are included, in addition to duplicates of 10 other components.** b**Mean total Cd (%) is the average total Cd content of all items in the respective categories.				

**Figure 3:**
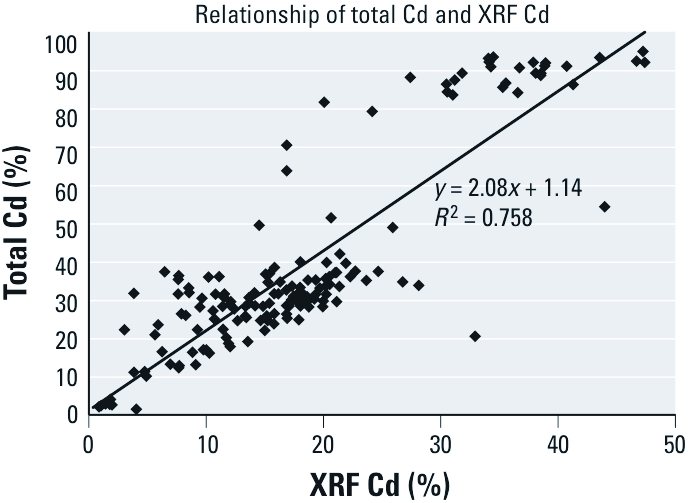
Correlation of total Cd content (as determined by total digestion and subsequent AA analysis) and Cd content as measured by XRF.

*Correlation of XRF analysis with total Cd content.* XRF is a surface analytical method and thus may yield results that differ from complete digestion of an item when surface platings are present. The correlation of XRF Cd with total Cd is shown in Figure 3. In general, total Cd content was approximately twice the amount indicated by XRF. The overall average Cd content of these samples was 18.30% by XRF and 39.27% by total digestion. XRF Cd was not strongly correlated to either saline-extractable (*r*^2^ = 0.41) or dilute HCl-extractable (*r*^2^ = 0.29) Cd.

## Discussion

Our data show that inexpensive high-Cd jewelry may release substantial quantities of Cd if mouthed or swallowed, although the amounts released vary greatly from item to item for reasons that most likely depend on surface platings and alloy composition.

*Saline-extractable Cd.* The 6-hr saline extraction yielded up to 2,200 µg Cd, and 10 of the 48 samples tested exceeded 18 µg saline-extractable Cd (Table 1). There was no correlation between the total Cd content of items and saline-extractable Cd. The two samples yielding the highest amount of saline-extractable Cd (2,109 and 2,189 µg) were duplicates of a football pendant with the highest total Cd content of any item tested (87.2 and 89.2% Cd, respectively). However, the samples that gave the next highest amounts of saline-extractable Cd (397 and 296 µg) were charms from the same charm bracelet that had the two lowest total Cd concentrations of samples tested, at 1.45 and 1.68%, respectively. Where duplicates were tested, item-to-item variability was often significant. Of particular concern is the fact that duplicates of two charms from a child’s bracelet yielded saline-extractable Cd both above and below the 18-µg threshold recommended by the CPSC.

*Dilute HCl-extractable Cd.* Previously, extraction of jewelry samples in 0.07 M HCl to determine bioavailability of metals has been used to evaluate the potential risks of highly leaded children’s jewelry (U.S. CPSC 2005a, 2005b). Under interim enforcement guidelines in place until the passage of the Consumer Product Safety Improvement Act (CPSIA), the CPSC stipulated that children’s jewelry could not both contain > 0.06% (600 ppm) total lead and yield > 175 µg of dilute HCl-extractable lead over 6 hr (U.S. CPSC 2005a). Whether 6 hr is an appropriate length of time for this test may be questioned, given that the residence time in the stomach for swallowed objects can be highly variable. Macgregor and Ferguson (1998) reported on 58 instances of children swallowing foreign objects in which the transit time through the digestive tract was measured. The transit time varied from 1 to 46 days, with a median of 6 days. The Macgregor and Ferguson paper was cited by the U.S. CPSC (2010e) as support for a longer (24-hr) extraction time for Cd-containing jewelry.

Our data show that HCl-extractable Cd increased linearly over 6–96 hr, indicating the potential for increasing harm the longer an item remains in the stomach (Figure 2) and supporting the CPSC recommendation for a 24-hr extraction time. However, replicate jewelry pieces of some items showed tremendous variability in dilute HCl-extractable Cd despite comparable total Cd composition (Figure 1), suggesting the importance of doing replicate testing of items. At 24 hr, 31 of 104 undamaged samples leached more than the CPSC-recommended threshold of 200.0 µg Cd, and the maximum amount of Cd released in 24 hr exceeded 20,000 µg. As was the case for saline-extractable Cd, there was no clear correlation between total Cd content and HCl-extractable Cd, consistent with the results of Streicher-Porte et al. (2008).

*Effect of damage to jewelry on bioavailable Cd.* A May 2010 petition to the CPSC and U.S. EPA regarding Cd in consumer products (Braiman et al. 2010) requested that the CPSC require that metal jewelry be cut in half before bioavailability extractions, to avoid misleading results from thin plastic coatings that would be damaged in normal wear by children. Electroplated coatings might also have a similar protective effect. Our data, based on a 1- to 2-mm cut in jewelry items, showed that the effect of damage was item-specific. In some cases, intact items leached more than damaged ones, both in the saline and dilute HCl extractions. In other cases, damage greatly increased Cd release. For the sandal charms used in the study of dilute HCl-extractable Cd over time, six damaged charms yielded > 30 times as much Cd (mean = 30,600 µg) as six intact charms (mean = 912 µg) over 96 hr. These results imply that simulation of realistic use is important if such bioavailability testing is the basis for regulatory compliance.

In summary, our data show that high-Cd jewelry can be a source of dangerous exposures to Cd for young children, given the bioaccumulative and highly toxic nature of Cd. The maximum amount of Cd leached by undamaged jewelry items exceeded the proposed CPSC limits for saline- and dilute HCl-extractable Cd by > 100 times. Further, these data show that variation in Cd bioavailability for replicate items can be quite high and that damage which breaches the outer surface of items can affect bioavailability, which will complicate assessment of compliance based on bioavailability measurements.
